# The development of an intervention programme to reduce whole-body vibration exposure at work induced by a change in behaviour: a study protocol

**DOI:** 10.1186/1471-2458-7-329

**Published:** 2007-11-15

**Authors:** Ivo JH Tiemessen, Carel TJ Hulshof, Monique HW Frings-Dresen

**Affiliations:** 1 Coronel Institute of Occupational Health, Academic Medical Centre (AMC), Meibergdreef 9, PO Box 22700, 1100 DE, Amsterdam

## Abstract

**Background:**

Whole body vibration (WBV) exposure at work is common and studies found evidence that this exposure might cause low back pain (LBP). A recent review concluded there is a lack of evidence of effective strategies to reduce WBV exposure. Most research in this field is focussed on the technical implications, although changing behaviour towards WBV exposure might be promising as well. Therefore, we developed an intervention programme to reduce WBV exposure in a population of drivers with the emphasis on a change in behaviour of driver and employer. The hypothesis is that an effective reduction in WBV exposure, in time, will lead to a reduction in LBP as WBV exposure is a proxy for an increased risk of LBP.

**Methods/Design:**

The intervention programme was developed specifically for the drivers of vibrating vehicles and their employers. The intervention programme will be based on the most important determinants of WBV exposure as track conditions, driving speed, quality of the seat, etc. By increasing knowledge and skills towards changing these determinants, the attitude, social influence and self-efficacy (ASE) of both drivers and employers will be affected having an effect on the level of exposure. We used the well-known ASE model to develop an intervention programme aiming at a change or the intention to change behaviour towards WBV exposure. The developed programme consists of: individual health surveillance, an information brochure, an informative presentation and a report of the performed field measurements.

**Discussion:**

The study protocol described is advantageous as the intervention program actively tries to change behaviour towards WBV exposure. The near future will show if this intervention program is effective by showing a decrease in WBV exposure.

## Background

It has been estimated that 4 to 7% of all drivers in some European countries, the USA and Canada are exposed to potentially harmful whole body vibration (WBV). Long-term occupational exposure to WBV is associated with an increased risk of disorders of the lumbar spine and the connected nervous system. The social and financial burden of disease from these disorders can be considerable. Only in the Netherlands 9% of the total days of sick leave under the working people can be attributed to LBP, 14% of all people who are unable to work, suffer from LBP and the total costs are estimated on 337 million euros [1].

In several European countries, it was therefore decided to recognize and compensate LBP and certain spinal disorders as WBV-related occupational diseases, as long as they meet certain criteria [2]. After many years of debate and preparation, a new European Directive on the protection of workers against the risks of vibration was published in 2002 and implemented in the national legislation of the EU member states in 2005. This Directive establishes minimum requirements for the protection of workers from risks to their health and safety that arise from exposure to mechanical vibration.

In line with this European Directive a multi-centred study, VIBRISKS, has been carried out, financed by the European Commission to prioritise research in the field of vibration exposed workers [3]. The goal of this VIBRISKS study is to improve understanding of the risk of injury from WBV at work by means of multi-national epidemiological studies supported by fundamental laboratory research. One of the objectives in this VIBRISKS study is to improve the understanding of the benefits of interventions like health surveillance by identifying factors that may result in the prevention of vibration-related symptoms or disorders. To fulfil this aim, we will develop an intervention programme at the workplace by using knowledge, own experience and previously described intervention programmes aiming to reduce WBV exposure.

A recent review by Tiemessen et al. [4] described that the determinants of WBV exposure can be categorized in two categories: 1. (technical) design considerations and 2. skills and behaviour. The emphasis in recent research is on the first category, though implications (especially based on behavioural changes) from the second category might be more promising in reducing WBV exposure at work.

Therefore, we developed an intervention programme with the aim to reduce WBV exposure in a population of drivers with the emphasis on a change in behaviour of driver and employer. The hypothesis is that an effective reduction in WBV exposure, in time, will lead to a reduction in LBP as WBV exposure is a proxy for an increased risk of LBP.

## Methods/Design

### Intervention strategy

A recent review by Tiemessen et al. [4] revealed a lack of evidence of effective intervention programmes to reduce vibration exposure on drivers. Although research on development of such programmes and their effectiveness is lacking, previous research makes it possible to describe the most important determinants causing WBV at work. The determinants are divided in two categories [4]. The first category concerns technical implications as type of seat, seat suspension or quality of the road or terrain and the latter category focuses more on the behavioural aspects towards WBV exposure. Although research concerning technical implications is still important, application of suspended seats alone is generally not sufficient to prevent drivers from harmful WBV exposure. In the literature, several authors recommend the adoption of a participative and multifaceted approach as a necessary feature of an intervention strategy to address the factors involved in the prevention and control of work-related musculoskeletal disorders [5,6]. Therefore, to make a reduction in WBV exposure to the drivers possible, both categories should be present in a intervention programme. However, the emphasis of this intervention programme, in contrast to recent research concerning WBV exposure, is on the second category (skills and behaviour). Determinants within the skills and behaviour category also have a large impact on WBV reduction and might be cheaper and/or more easily to implement. We therefore developed an intervention programme which is focussing on:

• A participative approach with a role for the driver, the employer, and the occupational health professional

• A multifaceted approach with factors concerning both design considerations and skills and behaviour, aiming at WBV reduction, however, with the emphasis on factors within the latter category.

We developed an intervention programme to change the behavioural aspects towards WBV exposure in accordance with the, in the field of health education often used, ASE model [7]. This ASE model can be used to explain attitude and attitude changes. The ASE model differentiates three cognitions: Attitude (A), Social influence (S) and Self- Efficacy (E). The model interprets human behaviour as reasonable, in other words humans know why they behave like they do. The model postulates that intention predicts behaviour. Together these three cognitions determine the motivation for certain human behaviour, the intention. The ASE model therefore is used to develop an intervention programme in order to change the attitude towards WBV which could result in a reduction of WBV exposure at the workplace. This change may include a reduction in the magnitude of vibration exposure received by the drivers and an increased recognition of symptoms related to vibration exposure.

### Attitude

According to the ASE model, we can divide the cognition attitude in affective and cognitive consequences of a certain behaviour. The emphasis of the intervention programme in this cognition of the ASE model is on the cognitive consequences. A substantial part in the intervention programme aims on increasing the knowledge of the driver and the employer. The affective consequence of the behaviour of the driver and also the employer is expected only to be altered when anticipated affective impediments of these stakeholders will be discussed in a personal approach. Therefore, at least in part, the intervention programme is participative with a role for the driver, the employer, and the occupational health professional.

### Social Influence

According to the ASE model, the cognition social influence is determined by the social standards on the work floor, the perceived behaviour of others and the social support for certain behaviour. The social standards on the work floor are expected to be affected when the drivers are more aware of exposure to WBV. Consequently, they will more easily accept a certain change in behaviour towards WBV exposure. A change in company policy towards WBV exposure will have it effects on the social standards on the work floor as well. The employer is responsible for this change in policy. It is expected that the perceived behaviour of co-workers will be influenced in the same order as the increase in awareness will change the social standards. Due to an increase in knowledge, WBV exposure becomes an issue at the work floor. The main change in the social support is expected by the role of the employer: the employer is expected to enable the driver to change its behaviour towards WBV exposure.

### Self-Efficacy

From the cognition self-efficacy three components can be distinguished: behaviour expectations, social expectations and strain expectations. Behaviour expectation is defined as the expectation of the driver to carry out a certain desired behaviour. Social expectation is defined as the expectation of (in this case) the driver to resist against social pressure from colleagues not to show the desired behaviour. Strain expectation is defined as the expectation of the driver to continuously show the desired behaviour. Behaviour expectations are expected to be affected by the role of the driver. By discussing or reflecting on the possible changes in behaviour to reduce WBV exposure, the driver may get the feeling he can actually do something about WBV exposure. The social expectation plays a minor role in the intervention program. By creating the right working environment due to changes in policy towards WBV exposure at work, the driver might feel he can continuously show the desired behaviour. In this way, the strain expectations are expected to be affected.

### Determinants of exposure

On the basis of the literature and experiences in our earlier studies [4,8,9] we identified the most important determinants of exposure to WBV. The identified determinants were: track or terrain condition, driving speed, driving style, type of tires, inflation of the tires, cabin suspension, seat suspension and seat adjustment.

The contact between wheel and surface is the main source of WBV exposure on the different vehicles. Therefore the track or terrain conditions is a central element in exposure, but also a key target in prevention. Different studies [10-12] have found a significant reduction in the WBV exposure when smoothening the track or terrain conditions and some studies [13-15] even found a significant decrease in WBV exposure. Driving speed and driving style is another major determinant of exposure. In general, the higher the speed, the higher the vibration magnitude will be [15-17]. In practice, track conditions and speed have a reciprocal relationship. Bad track conditions will limit the speed. The fitted type of tires (massive, cushion, pneumatic) can also influence the exposure [15,18], but the magnitude of this effect is in general less than that of track or speed. Little is known about using cabin suspension in vehicles, however, one field study [19] and a few laboratory studies [20-22] show promising results in decreasing the vibration magnitude when using the correct cabin suspension. Seat suspension may be considered as the last level of protection of the driver. In vehicles it is often the only level of protection used. Though, most research is performed on seat suspension. Despite some studies [13,23] who did not find any effect on the vibration magnitude when changing seat suspension, most studies report a positive effect (reduction in vibration magnitude) when using the correct seat suspension [10,15,24,25]. This correct choice of seat suspension is important, as every seat suspension is characterized by a cut-off frequency above which it attenuates the vibration. Below this value, it exhibits amplification of the vibrations. Furthermore, proper adjustment of suspended seat to the weight of the driver is essential for its correct working. In practice, this is often neglected.

These determinants are the basis of the intervention programme. The modification of one or more of these determinants will have a reducing effect on WBV exposure (for instance, by setting a limit to the driving speed (15 km/u)). To make a modification possible, agreements have to be made both with the driver and the employer.

### Operationalization of the figure for the drivers

**Figure 1:**
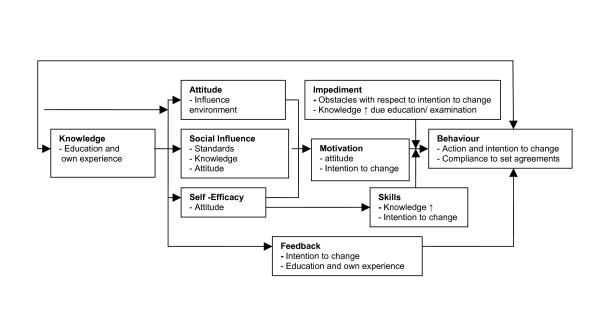
Figure of aspects according to the ASE model to change behaviour or the intention to change behaviour of the drivers with respect to WBV exposure.

The ASE model for the driver is represented in figure 1. From the starting point, knowledge is related to the attitude, the social influence and the self-efficacy of the driver. With providing an information brochure, an informative presentation and a newsletter, the knowledge of the driver is expected to increase. More specifically, the drivers will be provided with information about the newly implemented European Directive, legislation concerning WBV issues, the most important determinants of exposure to WBV and practical solutions to decrease WBV exposure. Besides increasing knowledge and changing attitude of the driver by providing the driver with information, an active approach to convince the driver to change his behaviour towards WBV exposure at work seems necessary [26]. For this active approach an individual meeting with an occupational health physician seems sufficient. In this meeting concrete agreements to decrease WBV exposure will be formulated. According to the figure, consequently, a change in attitude, social influence and self-efficacy will have its effect on the motivation of the driver to change or at least intent to change its behaviour towards WBV exposure. Furthermore, a change in self-efficacy has a direct influence on the skills of the driver by an increase in self-esteem and this will motivate the driver on his turn to change his behaviour concerning WBV exposure. This change in behaviour or the change in the intention to change behaviour can be diminished if impediments occur. Therefore, this intervention programme is participative, with a role for the employer as well.

### Operationalization of the figure for the employer

**Figure 2:**
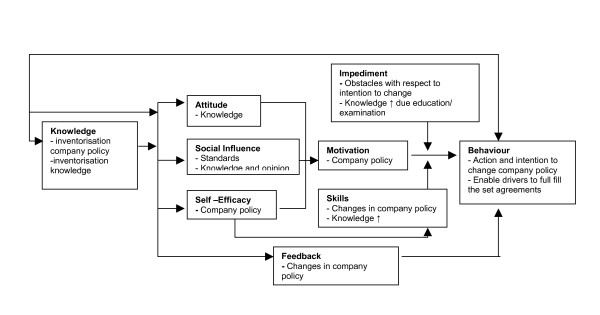
Figure of aspects according to the ASE model to change behaviour or the intention to change behaviour of the employers with respect to WBV- exposure.

The role of the employer in this intervention programme is bipartite. One, the role of the employer is to enable the drivers to change their behaviour or their intention to change their behaviour by taking away possible impediments such as: no means for innovations/investments concerning the vehicles of the drivers or not giving the driver the possibility to take mini-breaks because of the amount of work. The elaborated agreements formulated at the individual meeting between the driver and the occupational health physician will be discussed between the employer and the principal investigator to see if impediments exist. Second the employer is responsible for the company policy (i.e. purchase of new vehicles or seats, improving seat suspension or terrain conditions) towards WBV exposure at work which is in accordance with the newly implemented European Directive. The ASE model for the employer is represented in figure 2. From the starting point, knowledge is related to the attitude, the social influence and the self-efficacy of the employer. With providing an information brochure, an (individual) informative presentation and a general report concerning the field measurements in the company, the knowledge of the employer is expected to increase. This will, on its turn affect the cognitions: attitude, social influence and self-efficacy which are the determinants of the intention to a certain change in behaviour. With this change in behaviour in the employer, the impediments to an actual change in behaviour in the driver can be taken away and the policy towards WBV exposure at work might change.

### Outline of the programme

This operationalization results in the outline of the developed intervention program. The intervention programme consists of 4 different parts; (1) an individual health surveillance (2) an information brochure (3) an informative presentation and (4) a report concerning the results of the performed field measurements. At the start of the study all participants, both employers and employees (drivers), have given their written informed consent. The medical ethical committee considered that for this intervention study a full ethical approval procedure was not required because of the nature of the intervention (an approach to influence the vibration exposure) and the fact that the used intervention isbased on the Dutch Labour Conditions Act and the European Directive on Vibration (which have formulated obligations and procedures for employers and exposed employees).

### Health surveillance

**Figure 3:**
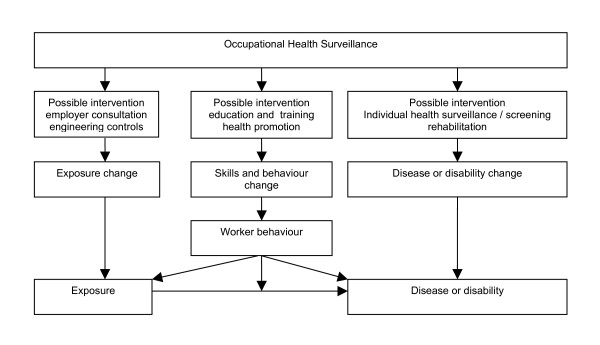
Figure of the concept how occupational health surveillance leads to an intervention.

Occupational health surveillance can be defined as 'the ongoing, systematic collection, analysis, interpretation and dissemination of data for the purpose of prevention' [27]. Surveillance is seen as essential to the planning, implementation and evaluation of occupational health programmes and control of work-related ill health and injuries and the protection and promotion of workers' health. Occupational health surveillance can be divided into workers' health surveillance and working environment surveillance [28,29]. In any case, health surveillance should lead to an intervention. Figure 3 illustrates this broad concept of occupational health surveillance [30].

The emphasis of health surveillance in this intervention programme was on the two left columns of the figure presented above. It was mainly an individual, one on one approach between the driver and the (trained) occupational health physician in which the actual complaints played a minor role. The emphasis of the health surveillance was on providing information about WBV exposure at work to the driver and to convince the driver that he can actually do something about this WBV exposure by changing his behaviour. The one on one approach was necessary as in this way the health surveillance was tuned more specifically to the driver (as each driver works in its own specific environment, with his own machine and his own specific working duties). The aim of the health surveillance was to increase the awareness of the risks of WBV exposure to the drivers and to set agreements to make a reduction in WBV exposure to the specific driver possible. The agreements made were based on the most important determinants of exposure to WBV and were also agreed with the employer of the driver. The health surveillance only takes place for the drivers which were exceeding the EU-action value (0,5 m/s^2^).

### Information brochure

Informative brochures were developed for the drivers as well as for the employers. These informative brochures aimed at: increasing knowledge and giving insight into practical solutions to reduce vibration exposure. The information brochure of the drivers and the information brochure of the employers were different but comparable with respect to the content. The employers brochure provided more explicit information about employers' responsibilities regarding WBV legislation, WBV policy and suggested preventive measures concerning WBV exposure. The information brochure will be send to all drivers in the intervention programme and to all employers of the different companies.

### Informative presentation

An informative, face-to-face presentation on the new WBV legislation and about possibilities for prevention was developed for both drivers and employers. The informative presentation has the same aims as the information brochure: increasing knowledge and giving insight into practical solutions to reduce vibration exposure both for the driver and the employer. Differences in the informative presentation to the drivers and to the employers are comparable with those in the brochures. By participating actively during the informative presentation, the drivers as well as the employer are subjected to the same information once again. It is known that by repetition, new information will be remembered better and will therefore increase the knowledge more permanently. The informative presentation only takes place for the drivers who are exceeding the EU- action value and their employers.

### Report of the performed field measurements

**Figure 4:**
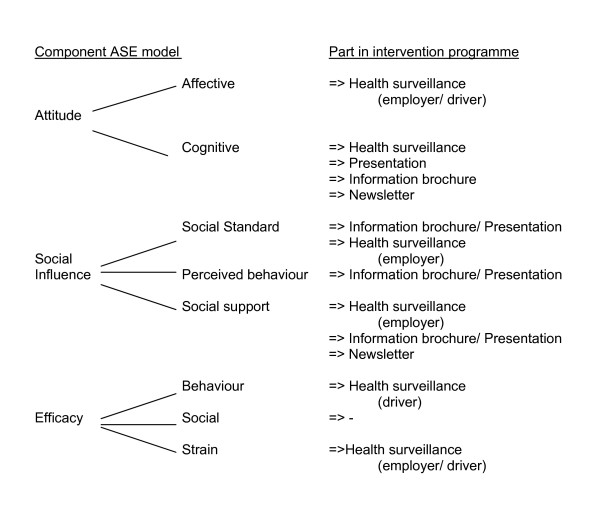
The representation of the components of the ASE model in the intervention programme for the driver and the employer.

The results of actual performed field measurements of the WBV magnitude will be send in the form of a standardized newsletter to the drivers and in the form of a general report to the employers. The aim of this newsletter or general report was: increasing awareness of the actual WBV exposure to which the drivers are exposed driving different kind of vehicles within the different companies. The newsletter will be send to all drivers in the intervention programme and the general reports to all employers of the different companies, (Figure 4).

### Application of the intervention programme

#### Population

From recent field measurements it can be concluded that a substantial part of the drivers of industrial vehicles in the Netherlands is exceeding the EU- action value (0,5 m/s^2^) stated in the European Directive. This implicates that these drivers are at risk of developing musculoskeletal disorders, with LBP in particular. Therefore, an intervention programme to reduce the WBV exposure will pre-eminently be suitable for drivers who are exceeding this action value. Drivers not exceeding the action level will also be included in the study but act as a control group. A change in WBV exposure under working circumstances involves a certain change in behaviour towards WBV exposure not only by the drivers but also by the employers. Therefore, both the drivers which are exceeding the action values and their employers play a specific role in the intervention programme.

#### Outcome measures for the driver

The main outcome variable of this intervention study is the reduction in WBV exposure. This WBV exposure is dependent of (a) the WBV magnitude and (b) the total time of exposure to this WBV magnitude based on an eight hours working day. The WBV magnitude will be measured at the workplace according to the requirements of the international ISO standard 2631-1 [31] and the VIBRISKS Protocol. The total time of exposure to this WBV magnitude will be assessed with the self-administered VIBRISKS questionnaire (Which of the following vehicles do you normally drive in the job, and for how many hours per week on average?). This VIBRISKS self-administered questionnaire [3] will be elaborated from an earlier version that was tested in several populations [32,33]. For each driver we will calculate the WBV exposure. Data of earlier studies and from data of existing WBV databases [34,35] will be used when the WBV magnitude is not actually measured.

Other outcome variables as: knowledge-, attitude- and behaviour scores for the drivers will also be assessed with a self-administered questionnaire before (T0) and after (T1) the implementation of the intervention programme. This self-administered questionnaire will be based on a standardized questionnaire used in a previous study from Hulshof et al. 2006 [9]. These questionnaires contain sections concerning attitude, social influence and self efficacy to come to positive and negative predictors of the behaviour of the driver and the employer. Knowledge will be assessed with a sum score of 22 questions (for instance: WBV are caused mainly by a bad track conditions or you can not do anything about back complaints yourself, answering options true or false). Attitude will assessed with a sum score of 11 questions (for instance: I take mini breaks often when I am performing my job or I perform exercises for my lower back muscles while I am at work, answering options never, sometimes, often and always). Behaviour will be assessed with 16 questions (for instance: Driving calmly in my job is impossible or A suspended seat is important for my health, answering options false, marginally false, marginally true, true).

Process variables as: the intention to change behaviour and the impediments of the intervention programme to become implemented will be asked with the T1 self-administered questionnaire with respectively, 15 and 10 questions. The feasibility of the targets and the compliance to the agreements which were set during the health surveillance, will be assessed by the principal investigator during a pre-structured telephone call. This pre-structured telephone call will be held after one month and will be repeated after 4 months. Compliance towards the intervention programme will be assessed during this pre-structured telephone call. The reasons for non-compliance will be asked in the T1 self-administered questionnaire.

#### Outcome measures for the employer

The main outcome measures for each employer are: the knowledge scores, the companies policy towards WBV and data about the impediments of the intervention programme to become implemented. Knowledge scores for the employers will be assessed with a comparable T0 and T1 self-administered questionnaire as the one for the drivers. Knowledge will be assessed with a sum score of 17 questions (for instance: The action value according the European Directive is?, answering options 0.3 m/s^2^, 0.5 m/s^2 ^or 0.8 m/s^2^). The company policy towards WBV, will be assessed with a sum score of 18 questions (for instance: Is there a policy in your company in driving time and resting time ratio, answering options yes or no). The impediments during the implementation of the intervention programme will be assessed and minimised during a meeting with the principal investigator. This meeting will take place after the health surveillance took place with the driver, to assure the agreements set in the health surveillance with the driver are agreed with the driver.

## Discussion

This study describes the development of a specific intervention programme aiming on a reduction of WBV exposure to drivers at work induced by a change in behaviour. To make a reduction in WBV exposure possible, we tried to change the behaviour or at least the intention to change the behaviour concerning WBV exposure of the drivers and the employers. In this intervention programme the specific role of the employer, next to taking his responsibility for the improvement of the working environment, was to enable the driver to change the behaviour concerning WBV exposure. Therefore, not only the driver but also the employer is involved. Because there is a lack of evidence on effective strategies to reduce WBV exposure, we had to rely on experiences gained in earlier epidemiological studies, on literature data and on incidental information or education material designed for different aspects of WBV. With the help of an earlier intervention programme [9] and a recently performed review [4], we identified the most important determinants of WBV exposure and included them in the intervention programme.

The design of this specific intervention programme has advantages. First, developing the intervention programme which is based on a change or at least an intention to change behaviour is favourable. This because, by changing behaviour the possible effect of the intervention programme is permanent (until the behaviour is changed again). Therefore, it has a long term effect and will work simultaneously with possible changes in WBV policy of the company. Another advantage of this intervention programme is that by changing behaviour towards WBV exposure, a possible effect in WBV reduction is realized without big investments. The determinants in the design considerations category, like introducing new seats or applying seat suspension can be very expensive, while driving more slowly or avoiding gaps in the track are relatively cheap. Third, by implementing this intervention programme not only the driver are responsible for their reduction in WBV exposure, also the employers have a specific role. This intervention programme implies certain changes in WBV policies. With this the whole company is aware of harmful exposure to WBV. Lastly, this intervention programme provides in an active approach of implementing preventive measures. This is in accordance with a study of van der Molen [15] who concluded that actively implementing preventive measures is an important facet of the success of an intervention programme. Passively providing the drivers and the employers an information brochure is not enough to establish a change in behaviour concerning WBV exposure. Instead by giving health surveillance on a individual basis in which the driver and the employer take place and interactive presentations to the employers and drivers a change in behaviour might be established.

Some aspects in the design of this specific intervention programme may need attention. One, when implementing this intervention programme, it is important to create a feeling by the driver that the employer has an important responsible role as well. Because the emphasis in this intervention programme is on the behavioural component of the driver, the role of the employer might be overlooked. Second, the follow-up time (7 months) in this study is short. However, as a decrease in WBV exposure is a proxy for a decrease in musculoskeletal disorders, the chosen follow-up time is expected to be sufficient to find a reduction in WBV exposure. Third, the intention to change a certain attitude should not be confused with the willingness to change a certain attitude. The literature shows that the willingness to change behaviour is conceptually different from the intention to change a certain attitude [26]. Willingness is thought to be reactive in nature and to be associated with little acceptance of the consequences of the new behaviour [36]. Intentions are thought to be related to such concepts as commitment, action and acceptance of the consequences of the new behaviour [16]. Employers and drivers, generally, are showing willingness to change a certain behaviour but only a small part of the employers and the drivers actually change their behaviour.

After implementation of this intervention programme, the effectiveness of this intervention programme will be evaluated. Success is depending on the main outcome: a decrease in WBV exposure. Process measures and indicators are formulated as well and process evaluation will make it possible to interpret the effect on the main outcome. A change in attitude or the intention to change attitude towards WBV exposure will hopefully contribute to a decrease in WBV exposure for drivers of various vehicles and LBP on the long term.

## List of abbreviations

LBP : Low back pain;

WBV: Whole body vibration;

VIBRISKS: Title of a multi-centred longitudinal study financed by the European Commission.

## Competing interests

The author(s) declare that they have no competing interests.

## Authors' contributions

MF, CH and IT conceived and designed the study. IT drafted the manuscript. CH obtained funding for this study. All authors read, made critical revisions and approve the final manuscript.

## Pre-publication history

The pre-publication history for this paper can be accessed here:


